# The relationship between social frailty and self-care ability in elderly stroke patients: the mediating role of self-efficacy

**DOI:** 10.3389/fpsyg.2026.1777204

**Published:** 2026-05-26

**Authors:** Xiaoyan Gong, Xue Hu, Mingjin Cai, Chun Xiao, Lingli Jiang, Yuan Ma

**Affiliations:** 1 Department of Neurosurgery, The General Hospital of Western Theater Command PLA, Chengdu, Sichuan, China; 2 Department of Oncology, The General Hospital of Western Theater Command PLA, Chengdu, Sichuan, China; 3 School of Nursing, Sichuan College of Traditional Chinese Medicine, Mianyang, China

**Keywords:** mediating and moderating effect, self-care ability, self-efficacy, social frailty, stroke

## Abstract

**Introduction:**

After stroke onset, patients commonly experience varying degrees of impairment in self-care ability, and psychosocial factors may play an important role in this capacity. This study aimed to examine the association between social frailty and self-care ability in elderly stroke patients and to explore the mediating role of self-efficacy in this relationship.

**Methods:**

A cross-sectional study was conducted in China between May 2024 and September 2025. A total of 436 elderly stroke patients were recruited. Data were collected on demographic characteristics, self-care ability as assessed by the Barthel Index, social frailty as measured by the Social Frailty Assessment Scale for the Elderly (SFASE), and self-efficacy as measured by the General Self-Efficacy Scale (GSES). Linear regression analyses were performed to examine the associations among social frailty, self-care ability, and self-efficacy. The mediating effect of self-efficacy was tested using the PROCESS macro in SPSS.

**Results:**

After controlling for demographic characteristics, social frailty was significantly and negatively associated with self-care ability (β = −0.211, *p* < 0.001), indicating that greater social frailty was associated with poorer self-care ability. Mediation analysis revealed a significant indirect effect of self-efficacy, with an effect value of −0.121. Self-efficacy partially mediated the relationship between social frailty and self-care ability, indicating that lower self-efficacy was associated with poorer self-care ability. Bootstrap mediation analysis showed that the direct effect (−0.090, 95% CI: −0.163 to −0.018) and the indirect effect (−0.121, 95% CI: −0.179 to −0.074) accounted for 42.76% and 57.24% of the total effect (−0.211, 95% CI: −0.285 to −0.137), respectively.

**Discussion:**

This study demonstrated that there is a significant negative correlation between social frailty and self-care ability in elderly stroke patients, and that self-efficacy partially mediates this relationship. The findings suggest that reducing social frailty and improving self-efficacy may be important targets in rehabilitation management, and provide a scientific basis for developing more individualized clinical intervention strategies.

## 1 Introduction

Stroke is primarily caused by the sudden rupture or occlusion of cerebral blood vessels and is characterized by abrupt onset and rapid progression, resulting in acute cerebrovascular events and brain tissue damage (Nadia et al., 2025). Stroke is currently the second leading cause of death worldwide. The World Stroke Organization projects that stroke-related mortality will increase by 50%, from 6.6 million (95% CI: 6.0–7.1 million) in 2020 to 9.7 million (95% CI: 8.0–11.6 million) in 2050 (Valery and Mayowa, 2023). At the same time, with the acceleration of global population aging, the number of older adults worldwide is expected to reach 2.1 billion by 2050, accounting for 22% of the global population (World Health Organization, 2023). Against this background, older adults have become the group most affected by stroke, making stroke an increasingly serious challenge not only for public health and healthcare systems, but also for healthy aging and functional independence in later life.

Epidemiological data from the World Health Organization (WHO) and the Global Burden of Disease Study indicate an important trend: although age-standardized incidence and mortality rates of stroke have shown a moderate decline, the absolute number of stroke cases among older adults worldwide has increased markedly because of the rapid growth of the aging population (Torres-Roman et al., 2025). After stroke, patients often experience persistent neurological deficits and incomplete functional recovery. Even after treatment, many individuals continue to experience varying degrees of motor impairment, cognitive dysfunction, language impairment, and other related difficulties (Takashi et al., 2023). Given its high prevalence, incidence, and disabling consequences, stroke has profound effects on quality of life, functional independence, and social participation in later life, making it a major challenge for healthy aging and long-term adaptation among older adults (Mei et al., 2025).

Functional independence is a core indicator of rehabilitation outcomes in stroke patients and directly affects quality of life, autonomy, and social reintegration in later life. Loss of self-care ability not only limits activities of daily living and may lead to disability, but also increases the risk of adverse complications, including pressure ulcers, pulmonary infections, and deep vein thrombosis (Samuelsson et al., 2020; Su et al., 2020; Dohle et al., 2024). Reduced independent living ability may further contribute to negative emotional outcomes, such as anxiety and depression (López-Espuela et al., 2020). Clinical data indicate that approximately 60%–80% of stroke survivors experience varying degrees of impairment in self-care ability after stroke onset (Sok et al., 2021; Tsivgoulis et al., 2023). Even after standardized rehabilitation treatment, some patients fail to regain their pre-stroke level of functioning and remain dependent on long-term care. This underscores the importance of examining factors related to the maintenance of self-care ability and adaptive functioning in older stroke patients (Feigin et al., 2016; Zhou et al., 2022).

With the growing emphasis on the biopsychosocial model of health, social frailty, as a geriatric syndrome parallel to physical frailty, has received increasing attention in aging research and in the prognosis of older adults with chronic diseases (Liu Q. et al., 2024). Social frailty is generally defined as decline in social functioning across domains such as social relationships, social support, and social engagement, and is specifically manifested in reduced social networks, insufficient social support, and financial difficulties (Fujiwara et al., 2022; Xiao-Ming et al., 2022). As an important psychosocial vulnerability in later life, social frailty may be particularly relevant to older stroke patients, whose physical dysfunction and cognitive impairment may restrict social activities and further reduce social participation (Zhang H. et al., 2025). Existing studies have shown that social frailty is closely associated with functional decline and poorer quality of life in older adults (Rojas-Avila et al., 2025). However, its relationship with self-care ability in older stroke patients remains insufficiently understood and warrants further investigation (Qiao et al., 2025).

Self-efficacy, derived from Bandura’s social cognitive theory, refers to an individual’s belief in their ability to successfully perform a specific behavior (Bandura, 1997). As a key social-cognitive construct, self-efficacy may play an important role in the psychological adaptation of older adults following stroke. In the context of stroke recovery, higher self-efficacy may motivate patients to actively engage in rehabilitation and maintain self-care behaviors (Aminu et al., 2021; Rasyid et al., 2023). Individuals with higher self-efficacy are more likely to cope proactively with condition-related challenges and to attempt activities of daily living independently, thereby facilitating the recovery of self-care ability (Szczepańska-Gieracha and Mazurek, 2020; Chau et al., 2021). Notably, social frailty may indirectly affect self-efficacy through its influence on patients’ psychological and social experiences. Individuals with insufficient social support and social isolation are more likely to experience loneliness and helplessness, which may in turn undermine their confidence in their ability to recover (Ma et al., 2018; Lee et al., 2024). These findings suggest that self-efficacy may serve as an important psychological mechanism linking social frailty to self-care ability in older stroke patients.

Previous studies have separately demonstrated the associations between social frailty and self-care ability, as well as between self-efficacy and self-care ability; however, the pathways linking these three factors remain insufficiently understood. More importantly, existing research has largely focused on pairwise associations, with limited attention to the specific psychosocial characteristics of older stroke patients or the psychological mechanisms underlying these relationships. Against this background, the present study was designed to systematically examine the status and interrelationships of social frailty, self-efficacy, and self-care ability in older stroke patients, with particular emphasis on testing the mediating role of self-efficacy. By doing so, this study aims to extend current understanding of psychosocial vulnerability, psychological adaptation, and functional maintenance in later life among stroke survivors. The following hypotheses were proposed: first, social frailty would be negatively associated with self-care ability in older stroke patients; second, self-efficacy would be positively associated with self-care ability and negatively associated with social frailty; third, self-efficacy would partially mediate the relationship between social frailty and self-care ability. The findings of this study may provide a theoretical basis for developing targeted intervention strategies to improve self-care ability in older stroke patients.

## 2 Materials and methods

### 2.1 Study design and sample

This cross-sectional study was conducted in Chengdu, Sichuan Province, China, between May 2024 and September 2025. Participants were recruited by convenience sampling from the General Hospital of the Western Theater Command of the People’s Liberation Army. The inclusion criteria were as follows: (1) a diagnosis of stroke confirmed by cranial computed tomography (CT) or magnetic resonance imaging (MRI); (2) basic verbal communication ability or the ability to understand the study purpose and questionnaire content through alternative communication methods, such as written text or gestures; (3) age ≥ 60 years; and (4) voluntary participation in the study. The exclusion criteria were as follows: (1) severe limb dysfunction, sensory impairment, or other physical conditions that could affect the assessment of self-care ability; (2) impaired consciousness or psychiatric disorders; and (3) the presence of other serious physical illnesses.

Data were collected by two trained researchers in outpatient clinics and hospital wards. The researchers explained the purpose and significance of the study to eligible stroke patients, and a standardized questionnaire was administered after voluntary informed consent was obtained. All participants completed the survey under the researchers’ guidance, and each questionnaire took approximately 20 min to complete. The data collection process followed the principles of informed consent, nonmaleficence, and confidentiality. Sample size was calculated *a priori* using G*Power 3.1 for multiple linear regression. Assuming a medium effect size (f^2^ = 0.10), an alpha level of 0.05, a statistical power of 0.90, and 14 predictors, the minimum required sample size was 415. After allowing for a 10% attrition rate, 480 participants were recruited. Of these, 436 valid cases were included in the final analysis (response rate: 90.8%), which was sufficient for the planned analyses. Specifically, 25 patients declined participation, 7 were excluded because of invalid questionnaire responses, and 12 were excluded because of concurrent severe cardiac, pulmonary, or hepatic diseases. Consequently, 436 stroke patients were included in this study. This study was approved by the Ethics Committee of the General Hospital of the Western Theater Command of the People’s Liberation Army (Approval No. 2022EC4-ky066).

### 2.2 Measures

#### 2.2.1 Demographic and clinical characteristics

The general information questionnaire was developed by our research team to collect data on demographic and clinical characteristics. The variables assessed in this study included age, gender, body mass index (BMI), smoking history, education level, marital status, mode of payment, employment status, place of residence, per capita monthly household income, history of surgery, and disease duration.

#### 2.2.2 Self-care ability

The Barthel Index is a standardized tool used to assess patients’ ability to perform activities of daily living. It was first developed by Mahoney and Barthel (1965). The index includes ten basic activities of daily living, with a maximum score of 100 points. A higher score indicates greater independence. Functional capacity is categorized into four levels based on the total score: 0–20 points indicates complete dependence; 21–60 points indicates severe dependence; 61–90 points indicates moderate dependence; 91–99 points indicates mild dependence; and 100 points indicates complete independence.

#### 2.2.3 Social frailty

The Social Frailty Assessment Scale for the Elderly (SFASE) was developed by Gong (2024) to assess social frailty. The scale consists of four dimensions: individual level (10 items), family level (8 items), social level (10 items), and societal level (8 items). It uses a five-point Likert scale, with scores ranging from 36 to 180, where higher scores indicate greater social frailty. A score above 115 denotes social frailty. Scores between 115 and 120 indicate mild social frailty, scores between 121 and 135 denote moderate social frailty, and scores above 135 signify severe social frailty. The scale has demonstrated excellent psychometric properties, with a Cronbach’s α coefficient of 0.926, split-half reliability of 0.928, test-retest reliability of 0.978, I-CVI ranging from 0.889 to 1, S-CVI/Ave of 0.930, and satisfactory construct validity (GFI = 0.813, TLI = 0.932, CFI = 0.937, RMSEA = 0.064). However, as it was developed recently, external validation and large-scale application studies of the scale are still limited, which should be considered when interpreting the findings.

#### 2.2.4 Self-efficacy

The General Self-Efficacy Scale (GSES) is used to assess self-efficacy and was developed by Schwarzer and Jerusalem (1995), Luszczynska et al. (2005). The GSES consists of ten items, each measured on a four-point Likert scale, with a total score ranging from 10 to 40 points. Higher scores indicate stronger general self-efficacy. Scores between 10 and 20 indicate a severe self-efficacy deficit, with individuals prone to withdrawal when facing challenges. Scores between 21 and 30 indicate moderate self-efficacy, reflecting reasonable confidence in familiar domains. Scores between 31 and 40 indicate strong self-efficacy, with individuals exhibiting full confidence when confronting challenges. In this study, the Cronbach’s α coefficient for the GSES was 0.87.

### 2.3 Statistical analysis

Data analysis was performed using IBM SPSS Statistics version 27.0. Categorical data were described using frequencies and percentages, while continuous data were summarized using means and standard deviations. Pearson correlation analysis was conducted to examine the relationships between social frailty, self-efficacy, and self-care ability, with a significance level set at α = 0.05. Linear regression was employed to investigate the relationships among social frailty, self-care ability, and self-efficacy, adjusting for demographic and clinical variables, including age, gender, body mass index (BMI), smoking history, education level, marital status, mode of payment, employment status, place of residence, per capita monthly household income, history of surgery, and disease duration. These covariates were selected based on their clinical relevance and evidence from previous studies on factors associated with social frailty and self-care ability in stroke patients. Model 1 included covariates; Model 2 included covariates and social frailty; Model 3 included covariates, social frailty, and self-efficacy. Mediation effects were examined using Model 4 of the PROCESS macro developed by Hayes for SPSS (Preacher and Hayes, 2008). To assess the direct, indirect, and total effects of the proposed model, bias-corrected bootstrapping was performed with 5,000 samples, evaluating results using 95% confidence intervals (CIs). An effect was considered statistically significant if its 95% CI excluded 0. Demographic and clinical variables were incorporated as covariates to control for mediating effects in the model.

## 3 Results

### 3.1 Participant characteristics

A total of 436 elderly stroke patients participated in this study, with a mean age of 74.32 ± 8.45 years. Regarding gender, 48.17% were male and 51.83% were female. In terms of body mass index (BMI), 28.44% had a BMI < 18.5, 58.03% had a BMI between 18.6 and 24, and 13.53% had a BMI > 24. With respect to surgical history, 43.12% of participants had a history of surgery, while 56.88% had no surgical history. Regarding disease duration, 55.28% of patients had a duration of ≤ 12 months, while 44.72% had a duration of > 12 months. Detailed participant characteristics are provided in Table 1.

**TABLE 1:** Demographic and medical characteristics of stroke patients (*n* = 436).

Variable	Categories	*N* (%)	Barthel Index score
Age	60∼75	244 (55.96)	70.94 ± 13.97
	≥ 76	192 (44.04)	60.81 ± 18.43
Gender	Male	210 (48.17)	67.50 ± 16.46
	Female	226 (51.83)	65.53 ± 17.17
BMI	< 18.5	124 (28.44)	63.87 ± 18.20
	18.6∼24	253 (58.03)	67.33 ± 16.17
	> 24	59 (13.53)	68.31 ± 16.20
Smoking history	No	224 (51.38)	68.10 ± 16.24
	Yes	212 (48.62)	64.76 ± 17.33
Education level	Junior high school or less	237 (54.36)	62.62 ± 16.63
	High school	176 (40.37)	71.02 ± 15.81
	College or higher	23 (5.28)	71.52 ± 16.97
Marital status	Single	40 (9.17)	61.00 ± 17.86
	Divorced/widowed/separated	187 (42.89)	64.14 ± 16.96
	Married/cohabitation	209 (47.94)	69.62 ± 15.93
Mode of payment	Self-paying	16 (3.67)	63.13 ± 14.88
	Health insurance	260 (59.63)	65.10 ± 17.65
	Agrarian insurance	160 (36.70)	69.06 ± 15.33
Employment status	Employed	165 (37.84)	66.21 ± 15.45
	Unemployed	80 (18.35)	61.63 ± 17.12
	Retired	191 (43.81)	68.74 ± 17.46
Place of residence	City and town	256 (58.72)	67.79 ± 15.80
	Rural	180 (41.28)	64.61 ± 18.09
Per capita monthly household income	< 3,000 RMB	226 (51.83)	65.93 ± 15.66
	≥ 3,000 RMB	210 (48.17)	67.07 ± 18.04
History of surgery	Yes	188 (43.12)	67.31 ± 16.47
	No	248 (56.88)	65.85 ± 17.12
Disease duration	≤ 12 months	241 (55.28)	69.56 ± 16.84
	> 12 months	195 (44.72)	62.67 ± 16.09

### 3.2 Correlations of social frailty, self-efficacy and self-care ability

The results for the scores and correlations of social frailty, self-efficacy, and self-care ability are presented in Table 2. The scores for social frailty, self-efficacy, and self-care ability were 70.36 ± 24.67, 28.03 ± 7.47, and 66.48 ± 16.86, respectively. Pearson correlation analysis revealed a significant negative correlation between self-care ability and social frailty (*r* = −0.414, *p* < 0.01), indicating that greater social frailty was associated with poorer self-care ability. A significant positive correlation was observed between self-care ability and self-efficacy (*r* = 0.493, *p* < 0.01), suggesting that individuals with higher self-efficacy demonstrated better self-care ability.

**TABLE 2:** Scores and correlation coefficients of the study variables (*n* = 436).

Variable	Mean ± SD	1	2	3
1. Social frailty	70.36 ± 24.67	1	–	–
2. Self-efficacy	28.03 ± 7.47	−0.340***	1	–
3. Self-care ability	66.48 ± 16.86	−0.414***	0.493***	1

SD, standard deviation. ***p < 0.001

### 3.3 Linear regression analysis of self-care ability

Linear regression analysis was performed to identify factors associated with self-care ability, with the results presented in Table 3. No multicollinearity issues were detected for any variables in this study (VIF < 5). After controlling for demographic and clinical factors, social frailty was found to have a significant negative correlation with self-care ability in Model 2 (β = −0.309, *p* < 0.001). When self-efficacy was added to Model 3 (β = 0.394, *p* < 0.001), the effect of social frailty on self-care ability decreased (β = −0.132, *p* = 0.015). This indicates that self-efficacy plays a crucial role in mitigating the negative impact of social frailty on self-care ability.

**TABLE 3:** Linear regression analysis of self-care ability (*N* = 436).

Variable	Model 1			Model 2			Model 3			VIF
	β	*t*	*P*	β	*t*	*p*	β	*t*	*P*	
Constant		13.018	< 0.001		14.596	< 0.001		9.623	< 0.001	
Age	−0.272	−6.066	< 0.001	−0.258	−5.935	< 0.001	−0.243	−6.125	< 0.001	1.078
Gender	−0.017	−0.389	0.697	0.008	0.187	0.851	0.012	0.299	0.765	1.055
BMI	0.028	0.641	0.522	0.006	0.13	0.897	0.024	0.61	0.542	1.065
Smoking history	−0.046	−1.039	0.299	−0.056	−1.303	0.193	−0.054	−1.385	0.167	1.037
Education level	0.167	3.704	< 0.001	0.137	3.131	0.002	0.118	2.956	0.003	1.281
Marital status	0.086	1.873	0.062	−0.007	−0.139	0.89	−0.001	−0.015	0.988	1.042
Mode of payment	0.124	2.809	0.005	0.103	2.417	0.016	0.128	3.279	0.001	1.046
Employment status	0.05	1.135	0.257	0.014	0.33	0.742	0.013	0.34	0.734	1.094
Place of residence	−0.09	−2.038	0.042	−0.104	−2.432	0.015	−0.105	−2.673	0.008	1.049
Per capita monthly household income	−0.043	−0.944	0.346	−0.118	−2.595	0.01	−0.11	−2.632	0.009	1.186
Surgery	−0.124	−2.737	0.006	−0.039	−0.833	0.405	−0.045	−1.061	0.289	1.038
Disease duration	−0.035	−0.785	0.433	−0.027	−0.636	0.525	−0.019	−0.498	0.619	1.226
Social frailty				−0.309	−5.609	< 0.001	−0.132	−2.454	0.015	1.983
Self-efficacy				0.260			0.394	9.228	< 0.001	1.246
R^2^	0.205						0.385			
Adjusted R^2^	0.182			0.237			0.364			

β, standardized beta.

### 3.4 The mediating role of self-efficacy in social frailty and self-care ability

**FIGURE 1:**
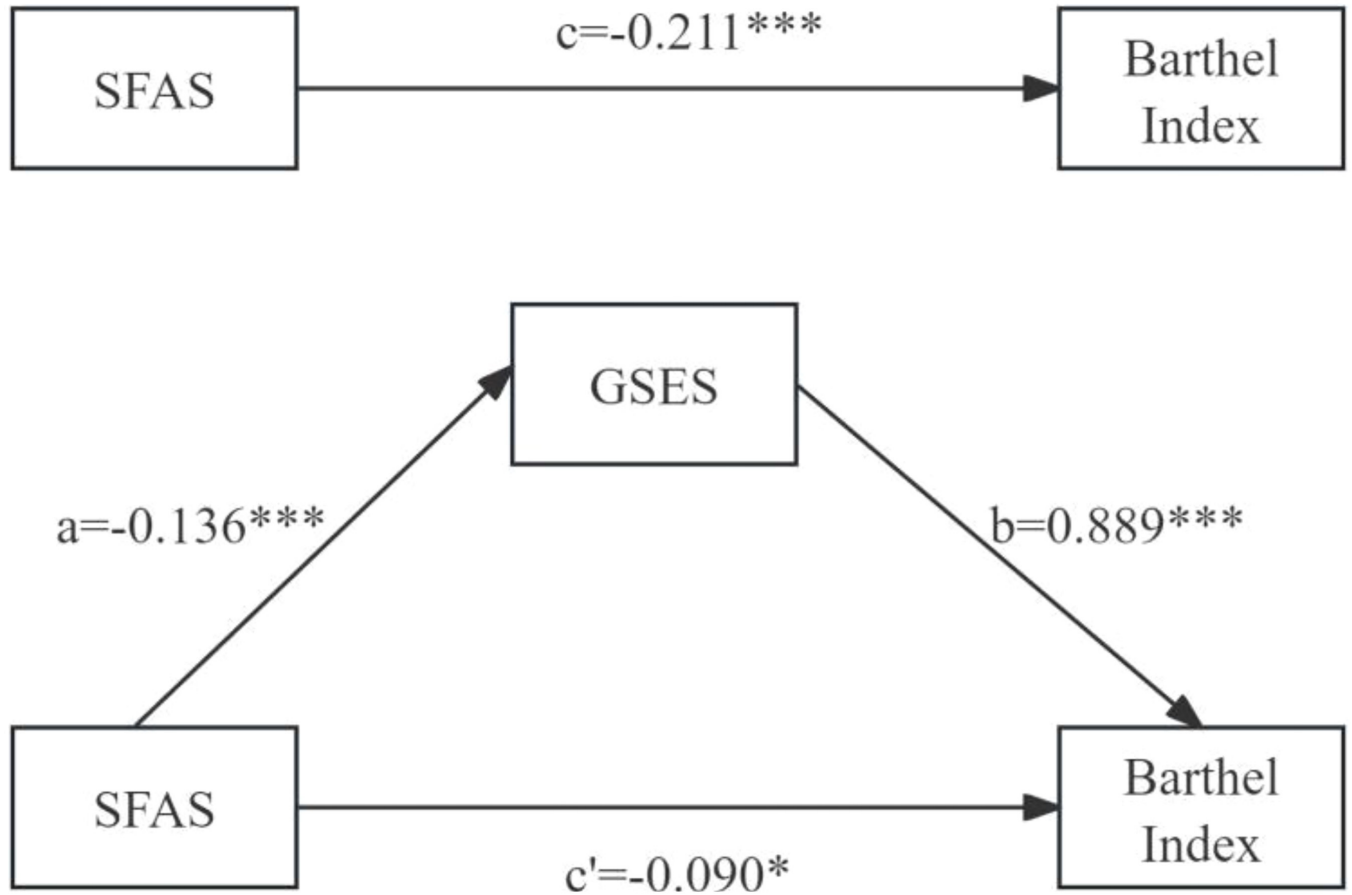
The mediating role of self-efficacy in social frailty and self-care ability. *p < 0.05, ***p < 0.001.

Mediation effects were examined using Model 4 within the SPSS PROCESS macro. Path coefficients for the three variables—social frailty, self-efficacy, and self-care ability—are illustrated in Figure 1. Initially, when examining the total effect of social frailty on self-care ability (the “c path”), a significant negative correlation was found between social frailty and self-care ability (c = −0.211, 95% CI: −0.285 to −0.137, *p* < 0.001). Mediation analysis revealed a significant indirect effect of self-efficacy, with a mediation effect size of −0.121 (95% CI: −0.179 to −0.074). Furthermore, after controlling for the mediating variable (self-efficacy), the direct effect of social frailty on self-care ability (c’ = −0.090, 95% CI: −0.163 to −0.018, *p* = 0.015) remained statistically significant, with the 95% confidence interval not containing 0. This indicates that self-efficacy partially mediated the relationship between social frailty and self-care ability. Table 4 reports the bootstrap mediation effect results, showing that the direct effect (−0.090) and mediating effect (−0.121) accounted for 42.76% and 57.24% of the total effect (−0.211), respectively. This analysis supports the hypothesis proposed in the introduction that self-efficacy is a significant factor in the relationship between social frailty and self-care ability. Specifically, the mediation model demonstrates that social frailty has a direct negative impact on self-care ability, but this effect is partially attenuated when self-efficacy is considered.

**TABLE 4:** Bootstrap mediation effect test results.

Effect relationship	Effect size	LLCI	ULCI	Effect proportion
Total effect	−0.211	−0.285	−0.137	
Direct effect	−0.090	−0.163	−0.018	0.428
Indirect effect	−0.121	−0.179	−0.074	0.572

## 4 Discussion

This study aimed to explore the mediating role of self-efficacy in the relationship between social frailty and self-care ability in elderly stroke patients. A total of 436 elderly stroke patients participated in the study, with a mean age of 74.32 ± 8.45 years. The average scores for social frailty, self-efficacy, and self-care ability were 70.36 ± 24.67, 28.03 ± 7.47, and 66.48 ± 16.86, respectively. The mediation model showed a significant negative correlation between social frailty and self-care ability, with self-efficacy playing a key partial mediating role. These findings support our hypothesis and provide empirical evidence for the underlying mechanism of impaired self-care ability in elderly stroke patients. This research offers new insights for developing targeted rehabilitation interventions and improving treatment outcomes in clinical practice.

The negative correlation between social frailty and self-care ability observed in this study is consistent with previous research (Makizako et al., 2015; Ma et al., 2018; Goto et al., 2024; Hamid et al., 2024). Elderly stroke patients often experience multiple functional deficits, such as impaired limb movement, balance, and cognitive abilities, due to brain tissue damage. Social frailty, as an early indicator of functional decline in older adults, includes manifestations such as reduced social participation, weakened social support networks, and loss of social roles. These factors contribute to the deterioration of external support systems, which are vital for patients’ rehabilitation (Liu M. et al., 2024; Zhang et al., 2024). Clinically, patients with limited social connections struggle to access sustained family support, community rehabilitation resources, and emotional encouragement. As a result, they may lack necessary assistance with daily activities such as dressing, personal hygiene, and eating, which can delay the recovery of their self-care abilities (Odaci Comertoglu et al., 2024). From a theoretical perspective, the lack of social support significantly influences the psychological and behavioral aspects of stroke patients’ rehabilitation. When patients experience social isolation or reduced social participation, their psychological resilience may be compromised. The absence of emotional support and encouragement can contribute to feelings of helplessness, anxiety, and depression, which can hinder motivation to engage in rehabilitation (Baek and Yoon, 2026). Additionally, a lack of social resources may prevent access to essential rehabilitation services, further exacerbating the decline in functional abilities. This interaction between social frailty and psychological functioning suggests that effective interventions should not only address physical impairments but also enhance the social and emotional support systems that are essential for a successful rehabilitation process (Li et al., 2023).

Data from this study reveal a significant positive correlation between self-efficacy and self-care ability in elderly stroke patients (*r* = 0.493, *p* < 0.001). This finding supports the applicability of social cognitive theory in geriatric stroke rehabilitation and highlights the crucial role of psychological factors in promoting physiological recovery (Gangwani et al., 2022). According to Bandura’s social cognitive theory, self-efficacy influences goal attainment by regulating cognition, motivation, and behavior. The restoration of self-care abilities in elderly stroke patients is a dynamic process involving physiological recovery, reinforcement of psychological beliefs, and consolidation of behavioral practices (Bandura and Cliffs, 1986). Cognitively, patients with high self-efficacy are more likely to view their abilities as improvable. They perceive rehabilitation challenges as obstacles to be overcome through effort, rather than as irreversible deficits. This cognitive tendency helps prevent learned helplessness (Wang T. et al., 2024). On a motivational level, self-efficacy influences patients’ expectations and persistence, enabling them to stay engaged in rehabilitation, even during prolonged or difficult recovery periods (Hess Engström et al., 2024). Despite setbacks, such as slow progress or repeated failures in daily activities, patients with higher self-efficacy are more likely to continue rehabilitation, avoiding the abandonment of self-care attempts due to frustration (D’Souza et al., 2024). Behaviorally, individuals with strong self-efficacy are more willing to explore personalized self-care strategies and demonstrate superior psychological resilience, leading to enhanced participation in rehabilitation programs and improved integration of motor function and self-care skills. This creates a positive cycle of enhanced self-efficacy, reinforced rehabilitation behaviors, and improved self-care capacity (French et al., 2016; Huang et al., 2025). These findings support the mediation hypothesis and underscore the importance of self-efficacy as a key target for psychological interventions in elderly stroke rehabilitation.

The core innovation of this study is confirming the partial mediating role of self-efficacy, revealing a dual pathway through which social frailty is associated with self-care ability in elderly stroke patients. On one hand, social frailty is associated with a reduction in external support resources (e.g., caregiving, rehabilitation, and emotional support), which may negatively impact self-care ability (Zhang Q. et al., 2025). On the other hand, social frailty is linked to a decrease in self-worth and confidence in recovery, which may undermine self-efficacy and indirectly hinder the restoration of self-care abilities. Self-efficacy, reflecting an individual’s confidence in their ability to perform specific tasks, is a key psychological factor in facilitating behavioral change (Rohde et al., 2025). Elderly stroke patients experiencing social frailty may be more vulnerable to negative emotions, such as loneliness and helplessness, which can lead to doubts about their ability to complete rehabilitation exercises and perform daily self-care activities (Riegel et al., 2021; Sardella et al., 2025). This diminished sense of self-efficacy can reduce motivation to engage actively in rehabilitation, potentially leading to the abandonment of self-care attempts and contributing to further decline in functional independence (Wang J. et al., 2024). These mediating effects suggest that, even in the presence of social frailty, interventions designed to enhance self-efficacy could help mitigate declines in self-care ability. This finding underscores the practical importance of focusing on self-efficacy in clinical rehabilitation interventions.

This study has several practical implications. In clinical interventions, healthcare professionals should adopt a comprehensive rehabilitation model that integrates physiological, psychological, and social factors. This model should not only focus on restoring motor function but also address social frailty. Strategies include organizing peer support groups, encouraging family involvement, and connecting patients with community-based social support resources (Yuan et al., 2021; Makino et al., 2024). Additionally, psychological interventions to enhance self-efficacy, such as cognitive-behavioral therapy (CBT), goal-setting techniques, and encouraging small, achievable goals, can significantly improve patient engagement in rehabilitation (Yildirim Üşenmez and Kavak Budak, 2026). These approaches foster a mindset that views rehabilitation challenges as obstacles to overcome, rather than permanent impairments, thus reinforcing patients’ belief in their ability to regain independence (Arghavan, 2017). In community health management, creating a database of social support networks for elderly stroke patients is essential. This helps provide targeted services like home-based care, community rehabilitation centers, and social activity platforms, especially for those at high risk of social frailty (Miao and Sau-Fung Yu, 2025). At the policy level, incorporating social frailty interventions into rehabilitation protocols can strengthen the integrated support system linking communities, hospitals, and households (Lu et al., 2023).

This study has several limitations. First, the cross-sectional design only allows for the examination of correlations between variables, preventing the establishment of causal relationships among social frailty, self-efficacy, and self-care ability. Therefore, longitudinal or interventional studies are needed to further explore these relationships. Second, the sample was drawn from a single healthcare institution using convenience sampling, which may introduce selection bias and limit the external validity and generalizability of the findings. Future research should include large, multicenter studies to validate and expand these results. Third, this study did not account for important psychosocial and clinical factors, such as depression, cognitive impairment, and caregiver burden, which could confound or moderate the relationships between social frailty, self-efficacy, and self-care ability. Including these variables in future multivariate models would provide a more comprehensive understanding of these relationships. Finally, the use of self-reported data may introduce response and subjective biases. Future studies could incorporate behavioral observation and objective measures to enhance the validity of the findings.

## 5 Conclusion

The findings of this study confirm that social frailty directly negatively affects the self-care abilities of elderly stroke patients, with self-efficacy playing a partial mediating role. Social frailty may indirectly diminish self-care capabilities by reducing self-efficacy. This outcome reveals the underlying mechanisms of self-care decline in elderly stroke patients from a socio-psychological perspective, highlighting the importance of fostering social support and enhancing self-efficacy in rehabilitation management. Future efforts should focus on developing a multidimensional rehabilitation model that integrates physiological, social, and psychological dimensions to enhance self-care abilities and improve the quality of life for elderly stroke patients.

## Data Availability

The original contributions presented in this study are included in the article/supplementary material, further inquiries can be directed to the corresponding authors.
